# Comparative purification and characterization of hepatitis B virus-like particles produced by recombinant vaccinia viruses in human hepatoma cells and human primary hepatocytes

**DOI:** 10.1371/journal.pone.0212800

**Published:** 2019-02-22

**Authors:** Edith Reuschel, Wolfgang Jilg, Birgit Seelbach-Goebel, Ludwig Deml

**Affiliations:** 1 Department of Obstetrics and Gynecology, University of Regensburg, Hospital of the Barmherzige Brueder, Clinic St Hedwig, Regensburg, Germany; 2 Institute of Medical Microbiology, University Medical Center, Regensburg, Germany; Academia Sinica, TAIWAN

## Abstract

This study describes the comparative expression and purification of hepatitis B surface antigen (HBsAg) particles produced upon infection of human primary hepatocytes and human hepatoma cell lines (HuH-7 and HepG2) with recombinant vaccinia viruses. The highest levels of HBsAg expression were found in HuH-7 hepatoma cells following infection with recombinant vaccinia viruses, which contain the S gene under control of a 7.5 k-promoter. Four different methods for purification of the HBsAg particles were examined: isopycnic ultracentrifugation, sucrose cushion sedimentation, isocratic column gel filtration, and binding to anti-HBs-coated microparticles. The highest degree of purity of HBsAg particles was reached by the method based on anti-HBs-coated microparticles. The resulting product was >98% pure. Biochemical analysis and characterization of purified HBsAg particles were performed by sodium dodecyl sulfate polyacrylamide gel electrophoresis (SDS-PAGE), western blotting, and electron microscopy. The HBsAg, purified from human hepatoma cell lines and from human primary hepatocytes, consisted of both the non-glycosylated (p25) and the glycosylated (gp27) form and assembled into typical 22-nm particles, and thus may be of great interest and importance for research, diagnostics, and medical treatments.

## Introduction

Hepatitis B virus (HBV) infection remains the cause of one of the most important infectious disease and represents a global health problem [1]. The worldwide number of chronic HBV-infections is estimated to be as high as 350 million people. Approximately 1 million death cases are attributable to acute and chronic HBV infection annually [2]. The World Health Organization (WHO) reports that approximately 65 million women are chronically infected with HBV which leads to a high risk of mother-to-child transmission [3]. Especially in endemic regions, vaccinations are still not affordable, for example in some areas in Africa where up to 10% of the population are chronic HBV carriers [4]. HBV infection is directly correlated with the risk of developing chronic hepatitis, progressive liver cirrhosis and hepatocellular carcinoma [5].

The best way to control the infection and to prevent vertical transmission is to use a vaccine. The first vaccines against hepatitis B virus were purified from serum of HBV infected chronic carriers [6] and consisted of adjuvanted hepatitis B surface antigen (HBsAg) particles. The limited supply of plasma from chronically infected humans and safety concerns demanded an alternative to plasma-derived particles serving as vaccines [7]. Since then, the S-gene has been expressed in many different systems, such as prokaryotic cells [8], yeast [9–14], stably transfected mammalian cells including mouse fibroblasts [15,16] and chinese hamster ovary (CHO) cells [17], mammalian cells infected with recombinant vaccinia viruses [18–22], insect cells infected with recombinant baculoviruses [23–25], and plants [26], in order to produce a sufficient amount of safe and effective HBsAg-based recombinant vaccine. Meanwhile, HBV surface antigen (HBsAg) lipoprotein particles are the basic components in almost all experimental and commercially used HBV candidate vaccine preparations. The pre-S-containing antigens are more immunogenic than vaccines only consisting of the S-gene products, namely the major polypeptide (p25) and its glycosylated form (gp27) [27]. All information to self-associate and mobilize cellular lipids into spherical lipoprotein particles with approximately 22 nm diameters is included in the small (S) proteins [28]. These S-particles have been clearly demonstrated to induce a protective antibody response against an HBV infection [29,30]. Furthermore, direct administration of plasmid DNA encoding the S-gene has been shown to induce HBsAg-specific humoral and cell-mediated immune responses [31,32]. Today, the commercially available efficient recombinant vaccines are based on HBsAg particles derived from yeast or Chinese hamster ovary (CHO) cells [33–35] and are relatively inexpensive to produce, safe, and well tolerated. Thus, there is no immediate necessity to replace these vaccines. On the other hand, in many developing countries, especially in Africa, there are still 3 vaccine doses necessary to provide effective protection against HBV infection. Additionally, 2.5% to 5% of healthy immunocompetent vaccine recipients, as well as many immunocompromised patients, do not respond well to the vaccines [36–38]. In recent years, HBV S-gene mutants affecting the "a" determinant [39–41] have been reported, as well as a few mutations outside this major immunodominant region [2,42]. Therefore, to extend vaccine protection to large populations of the "third world" and to hypo- or non-responsive-individuals, for example children with celiac disease [43], the evaluation of alternative vaccines against HBV and the search for second generation recombinant vaccines with the potential for increased protection is necessary.

The current manuscript describes the comparative expression, purification and biochemical characterization of HBsAg particles produced by recombinant vaccinia viruses in primary hepatocytes as a more physiological and not oncologically-altered model system compared to different hepatoma cell lines. The vaccinia virus system offers a fast, simple, and highly efficient strategy for the production of foreign antigens. Expression of correctly glycosylated HBsAg was observed in all tested cell lines. The best expression rates were observed in the human hepatoma cell line HuH-7. Different purification procedures based on simple methods, such as protein concentration, precipitation, ultracentrifugation, chromatography or specific particle binding were compared. The HBsAg particles purified from human liver cells are morphologically and biochemically similar to HBsAg particles derived from other mammalian expression systems. In contrast to yeast- and insect cell-derived HBsAg the HBs-proteins were correctly glycosylated and assembled into typical 22-nm particles. Thus, vaccinia virus expression in human primary liver cells provides an excellent alternative methodology for limited production of biologically active HBsAg particles for use in laboratory and diagnostic research.

## Materials and methods

### Tissue sources, isolation, and cultivation of primary hepatocytes

Human hepatocytes were prepared from non-neoplastic lobectomy segments resected from adult patients undergoing liver surgery at the University Medical Center of Regensburg for medically required purposes unrelated to our research program, and obtained via the charitable state-controlled foundation Human Tissue and Cell Research (HTCR, Regensburg, Germany). All experimental procedures were performed according to the guidelines of HTCR with the patients’ written informed consent approved by the local ethical committee of the University of Regensburg [44]. All experiments involving human tissues and cells were carried out in accordance with the "WMA International Code of Medical Ethics" [45]. Liver specimens were received within 24 h after surgery and shipped to the laboratory on wet ice. Shipping solution was HEPES-buffered Hank’s balanced salt solution (HBSS, pH 7.8, containing 1.78 mM of NaHCO_3_, 5.5 mM of D-glucose, 0.001% phenol red, 10 mM of HEPES, 0.1% methylcellulose of low viscosity) containing 10 U of penicillin, 10 ng/ml of streptomycin, 500 ng/ml of fungicide, and 5% fetal bovine serum (FBS). Biopsies of normal liver tissue, weighing 20 to 40 g, were transferred to calcium-free Krebs-Ringer buffer (KRB) at 4°C. The portal and hepatic veins of the liver segment were cannulated. The specimen was sequentially perfused with KRB for 10 min, followed by KRB containing 0.5 mg/ml of collagenase and 5 mM of CaCl_2_ for another 10 min. Type I collagenase solution was prepared by dissolving reagents (NaCl 8.0g/l, KCl 0.4 g/l, NaH_2_PO_4_ x H_2_O 0.078 g/l, NaH_2_PO_4_ x H_2_O 0.151 g/l, CaCl_2_ 0.56 g/l, HEPES 2.38 g/l, phenol red 0.006 g/l, trypsin inhibitor 0.05 g/l NaHCO_3_ 0.35 g/l) in deionized water (without collagenase), adjusting the pH to 7.5. Collagenase (0.5 g/l) was dissolved gradually, the solution was stirred for 1 h, and the pH was adjusted to 7.5. The collagenase solution was then sterilized by passing through a 22-nm filter. All solutions were equilibrated with 95% O_2_ and 5% CO_2_ at 37°C. The perfusion rate was continuously adjusted to maintain a pressure of approximately 102 +/- 10 mmHg. After perfusion, the cells were dispersed, filtered, and washed twice with cold KRB. Purification of the cell suspension was achieved using Percoll gradiation. Approximately 40 million viable hepatocytes were obtained per gram of liver. Hepatocytes were resuspended in the Dulbecco´s-modified Eagle´s medium (DMEM; Gibco-BRL, Karlsruhe, Germany), supplemented with 0.5 unit/ml of insulin, 1.07 ng/ml of glucagon, 7.5 μg/ml of hydrocortisone, and 0.02 μg/ml of epidermal growth factor (EGF) as the standard hormone composition. EGF was added to the culture medium by appropriate dilution to induce the S-phase of the cell cycle. Cells were seeded at 1.2 or 4 million cells per 60 x 15 mm plastic dish (Falcon, Franklin Lakes, NJ, USA) in a total volume of 2 ml of the above described complex medium. At least 30 min prior to plating, dishes were coated with a collagen gel layer (1.1 mg/ml type I collagen; Serva). A double gel configuration was performed by aspirating the cell-culture medium after 24 h of cultivation and applying the second layer of collagen. After a gelation period of 30 min, medium was added. Spent medium was replaced by fresh medium every 24 h in the absence of serum. The cells were incubated at 37°C in a humidified atmosphere, containing 5% CO_2_. For long-term infection experiments of the primary hepatocytes, 200 μl of the culture supernatant were removed daily and refilled by fresh medium. The supernatant was examined for HBsAg content by microparticle enzyme immunoassay (MEIA). Culture supernatant was collected each day until the 68th day of incubation.

### Cell cultures

The human hepatoma cell lines HuH-7, HepG2, and HepG3 were routinely maintained in DMEM, supplemented with 10% FBS and 1% (v/v) penicillin/streptomycin (Gibco-BRL). The constitutively HBsAg-expressing HepG3 cell line was grown in plastic cell culture flasks (75 cm^2^ in surface area, Becton Dickinson, Franklin Lakes, NJ, USA) using 12 ml of complete or serum-free DMEM at 37°C for the production of HBsAg particles.

### Recombinant vaccinia viruses

A recombinant vaccinia virus containing the coding region for the hepatitis B surface antigen (subtype ayw) was used. The construction of the HBsAg-recombinant vaccinia virus has previously been described in detail [46]. Briefly, a 1.8-kb *Xho*l/*Bgl*II HBsAg-encoding DNA fragment of plasmid pSHH 2.1 was inserted into a *Bgl*II/*Sal*I linker sequence 31 nucleotides downstream of the early RNA start site of the vaccinia 7.5 k-promoter flanked by a thymidine kinase sequence. Recombination and selection of HBsAg-expressing recombinants was performed following standard procedures [47–49]. Transient expression in CV-1 cells was performed using the recombinant vaccinia virus T7 system encoding T7 RNA polymerase [50,51]. Primary hepatocytes and hepatoma cells were transfected with 1 μg of purified cDNA and 9 μl of Lipofectamine (Gibco-BRL) according to the manufacturer´s protocol. Three hours after transfection, the cells were infected with vaccinia virus vTf7-3 (multiplicity of infection (M.O.I.) = 1) for 30 min at 37°C. After the medium was replaced by 2 ml of DMEM supplemented with 10% fetal calf serum, incubation was continued for 24 h. Then, primary hepatocytes or hepatoma cells were scraped off the plate into 250 μl of phosphate-buffered saline (PBS) containing 0.05% (vol/vol) tween 20, pelleted, suspended in 1.2 ml of PBS, and disrupted by 3 freezing-thawing cycles. After centrifugation at 14,000 g for 10 min, the supernatant was removed, and equal aliquots of the pellet were solubilized in the different buffers by shaking for 10 min at room temperature. The extracts were centrifuged as described above, and supernatants and pellets were analyzed by sodium dodecyl sulfate polyacrylamide gel electrophoresis (SDS-PAGE).

### Expression and quantification of HBsAg

Hepatoma cells or primary hepatocytes were infected with recombinant vaccinia viruses at multiplicities of 1 to 0.0001 M.O.I./cell. Hepatoma cells or primary hepatocytes were seeded in 6-well-plates or cell culture bottles and allowed to grow. Cell culture supernatant was harvested at different time points and clarified at 9,600 x g for 20 minutes at 4°C. Constitutively HBsAg-producing HepG3-cells were routinely cultured in 75 cm^2^ cell culture flasks.

The quantities of HBsAg were assayed from the cell culture supernatant by a commercial IMx HBsAg (V2) microparticle enzyme immunoassay (MEIA; Abbott Diagnostics, Wiesbaden, Germany; B2W280/67-6958R3; now discontinued and replaced by the Architect HBsAg Qualitative chemiluminescent microparticle immunoassay, B1P970/48-5882R2) according to manufacturer’s recommendations. Briefly, HBsAg was specifically captured on microparticles coated with (mouse monoclonal) anti-HBsAg antibodies and detected with biotinylated (goat) anti-HBsAg antibodies. These antibody-antigen-antibody complexes bound to the microparticles were then transferred and irreversibly bound to a glass fiber matrix. The matrix was incubated with a (rabbit) alkaline phosphatase-conjugated anti-biotin antibody, washed to eliminate unbound materials, and the fluorescent product (indicative of reactivity to HBsAg) was revealed by the addition of the substrate 4-methylumbelliferyl phosphate. The rate of formation of fluorescence product was measured by the MEIA optical assembly, in parallel to the Calibrator (IMx HBsAg (V2) MODE1 Calibrator, No. 2228–21) and to negative and positive controls (human plasma non-reactive and reactive for HBsAg, respectively; IMx HBsAg (V2) No. 2228–10). Results are expressed as signal-to-noise (S/N) values, where S/N is the ratio of the sample rate to the Calibrator rate. The cutoff rate is defined as the Calibrator rate x 2. A sample is considered reactive for HBsAg when its rate ≥ cutoff. Supernants were diluted so that the measured S/N values are within the linear range of detection, as determined by a 6-point calibration curve performed using purified yeast-derived recombinant HBsAg (not adjuvanted; kindly provided by GlaxoSmithKline, Rixensart, Belgium; [52]). The depicted S/N values were calculated by multiplying the measured S/N values by the respective dilution factor.

### SDS-PAGE and Western blot analysis

Purified and concentrated cell culture supernatants were denatured under reducing conditions at 100°C for 5 min in SDS loading buffer according to Laemmli [53]. Proteins were separated on 15% polyacrylamide gel and transferred by electroblotting to a nitrocellulose membrane. Individual proteins from identical polyacrylamide gels were visualized by silver staining. HBs-antigen was detected by western blot analysis using biotinylated (goat) polyclonal HBsAg-specific antibodies and a (rabbit) anti-biotin alkaline phosphatase conjugate for staining (Abbott IMx HBsAg (V2), Reagent Pack No.2228-21).

### Isocratic column gel filtration

Protein separation was performed by preparative isocratic gel filtration on a Superdex 200 prep grade column (HiLoad 16/60, Pharmacia, Uppsala, Sweden). Before use, the column was washed with one bed volume of buffer of low ionic strength with a flow rate of 25 cm/h (= 0.8 ml/min) to remove residual traces of ethanol. The column was then equilibrated with another 2 bed volumes of separation buffer. To remove proteins non-specifically adsorbed to the gel, the column was cleaned by washing with 0.5 to 1 bed volume of 0.5 M NaOH. Then, the column was immediately equilibrated with 2 bed volumes of separation buffer. HBsAg separation was performed with PBS flow buffer (pH 7.5) with a flow rate of 0.5 ml/min and a total column volume of 120 ml.

### Isopycnic KBr gradient fractionation

Cell culture supernatants of primary liver hepatoma cells were collected and centrifuged for 10 min at 10,000 rpm to exclude cell debris. Isopycnic banding by KBr (Merck, Darmstadt, Germany) gradient fractionation to equilibrium was carried out to purify particulate HBsAg. Following the removal of undissolved material by centrifugation at 20,000 g for 10 min, supernatant was filtered using an Amicon membrane (XM-300). Powdered KBr was added to the resulting concentrated solution to a final concentration of 24 to 26% (w/w). The sample was then ultracentrifuged in Quick-Seal tubes (Beckman) for 21 h at 60,000 rpm and 15°C using a Beckman Ti70 rotor. The linear gradient formed after KBr ultracentrifugation was fractionated, and the single fractions were measured by MEIA.

### Sucrose cushion sedimentation

HBsAg particles were purified from cell culture supernatant by sedimentation through a 20% sucrose cushion (5 ml sucrose solution overlayed with 30 ml cell culture supernatant). Subsequently tubes were ultracentrifuged at 25,000 rpm for 16 h using a Beckman SW-28 rotor. Supernatant was discarded, and pellets were suspended in PBS. HBsAg concentration was determined by MEIA.

### Purification by anti-HBs-coated microparticles

Cell culture supernatant was harvested at different time points and clarified at 2,860 x g for 20 min at room temperature to remove cell debris. Monoclonal (mouse) anti-HBs coated microparticles (Abbott IMx HBsAg (V2), Reagent Pack No.2228-21) were added (20 μl microparticle suspension per ml culture supernatant with a concentration of 1.3 μg/ml). The supernatant was incubated for 1 h at 37°C before centrifugation (2,860 g) for 40 min at 4°C. Pellet was transferred to a 1.5-ml tube and washed 3 times with 500 μl of H_2_O and once with PBS, followed by centrifugation for 10 min at 15,000 g. The resulting pellet was resuspended in H_2_O, sonified for 3 min and disrupted by 3 freezing-thawing cycles. Exclusion of anti-HBs-coated microparticles was carried out by adding 5 to 10 μl of 1 N HCl solution. After precipitation, the solution was transferred into a new tube, vortexed, and centrifuged for 15 min at 30,000 g and 4°C. Neutralization of supernatant was performed by adding 1 N of NaOH.

### Electron microscopy

As described by Osterrieder et al. [54], electron microscopy of HBsAg particles was performed. After fixation with 2% glutaraldehyde, purified particles were adsorbed to grids. Following 3 washes in tris-buffered saline (TBS), grids were contrasted with phosphoric tungstic acid and examined by electron microscopy.

## Results

### Expression of HBsAg-particles

Human primary liver cells, embedded between 2 collagen layers, as well as human hepatoma cell-lines (HuH-7, HepG2) were infected by HBsAg recombinant vaccinia viruses at different M.O.I.. At different time points after inoculation, ranging from 24 to 96h, cells were harvested, culture supernatant was clarified by low speed centrifugation, and the yields of secreted HBsAg were determined by commercial microparticle enzyme immunoassay (MEIA).

**Fig 1:**
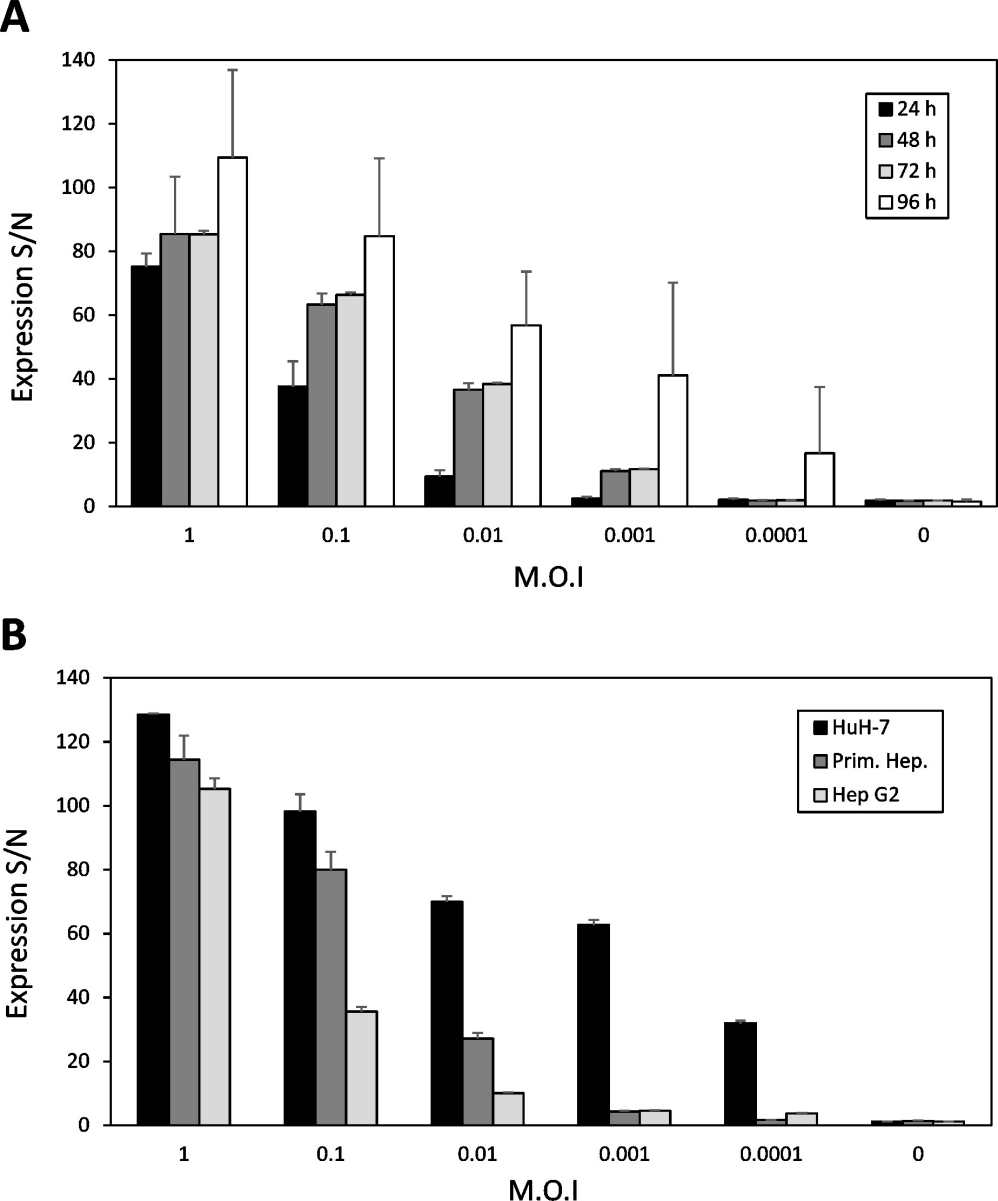
Expression of HBsAg particles by recombinant vaccinia viruses in different cell lines. (A) The hepatoma cell line HuH-7 was infected with various M.O.I. [multiplicity of infection] of HBsAg-recombinant vaccinia viruses. The expression level of HBsAg was measured from the cell culture supernatant (S/N-value) by an IMx microparticle enzyme immunoassay at indicated time points: 24, 48, 72, and 96 h after infection. (B) HuH-7 hepatoma cells, primary hepatocytes, and HepG2 were comparatively infected with recombinant vaccinia viruses at the indicated M.O.I´s. Four days after infection, cells were harvested, and the expression level of HBsAg was measured in the supernatant by an IMx microparticle enzyme immunoassay.

High levels of HBsAg expression were observed in all cell lines following infection with recombinant vaccinia viruses. The highest expression rates of HBsAg were detected in HuH-7 hepatoma cells with an M.O.I of 1. Expression rates increased over time and correlated with the M.O.I. used for infection (Fig 1A). Almost no reactivity of HBsAg expression was detectable in HuH-7 cells 24 h after infection with recombinant vaccinia viruses at an M.O.I. of 0.001 or lower. In the next set of experiments, we compared the expression of HBsAg of hepatoma cell lines and primary hepatocytes 96 h after infection (Fig 1B). Again, the decrease in HBsAg expression rates in HuH-7 cells and primary hepatocytes correlated with the decrease of M.O.I. of vaccinia viruses used in the infection experiments. Infected primary hepatocytes produced 5% to 10% lower amounts of HBsAg than HuH-7 cells. The most drastic decrease in HBsAg production, following infection with HBsAg-recombinant vaccinia viruses in correlation to the M.O.I., was found when comparing HepG2 cells to HuH-7 cells. The comparison of both hepatoma cell lines revealed a 0.5- to 3-fold decrease in HBsAg production from HuH-7-cells as compared to HepG2-cells.

**Fig 2:**
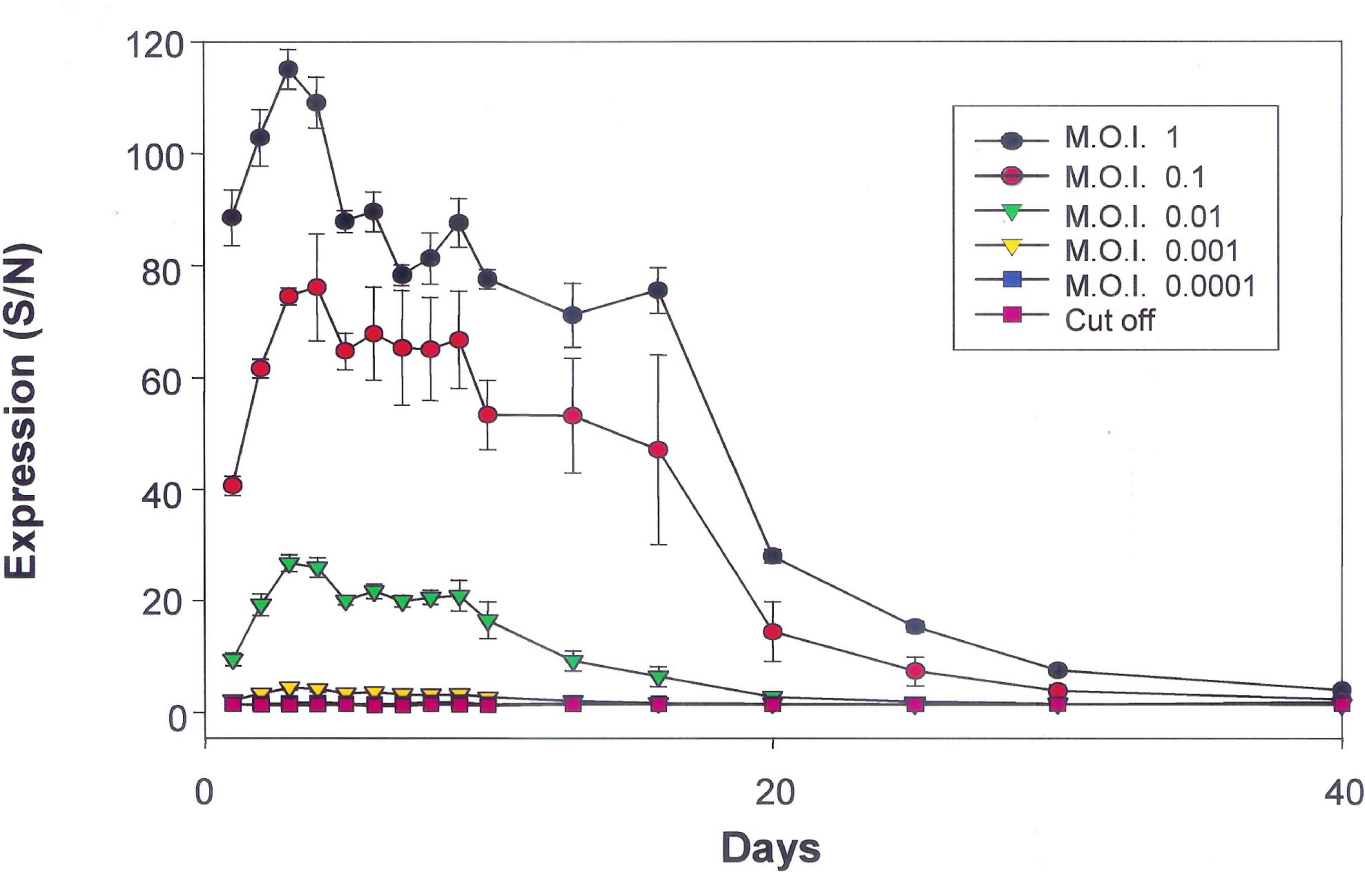
Long-term HBsAg-expression by human primary hepatocytes following infection with recombinant vaccinia viruses. Human primary liver cells were isolated by EGTA/collagenase-perfusion and cultivated between 2 collagen layers. After infection with HBsAg-recombinant vaccinia viruses, the HBsAg concentration was determined from the supernatant over a period of more than 40 days using an IMx MEIA test.

In further experiments, long-term HBsAg expression of human primary hepatocytes infected by HBsAg-recombinant vaccinia viruses was determined (Fig 2). Primary liver cells were infected with different M.O.I.´s of HBsAg recombinant vaccinia viruses and cultured over a time period of more than 40 days. A significant correlation between M.O.I. of vaccinia viruses and the amount of HBs antigen production was observed. The maximal rate of HBsAg secretion was reached at an M.O.I. of 1, 3 days after infection (115.1 S/N). In all 3 double gel cultures of primary hepatocytes, significant HBsAg concentrations were detectable up to 18 days.

### Comparison of different purification methods for HBsAg

In order to characterize the HBsAg produced, the particulate antigens were purified by different techniques, including isopycnic ultracentrifugation, isocratic column gel filtration, sucrose cushion sedimentation, and binding to anti-HBs-coated microparticles.

#### Isopycnic ultracentrifugation

**Fig 3:**
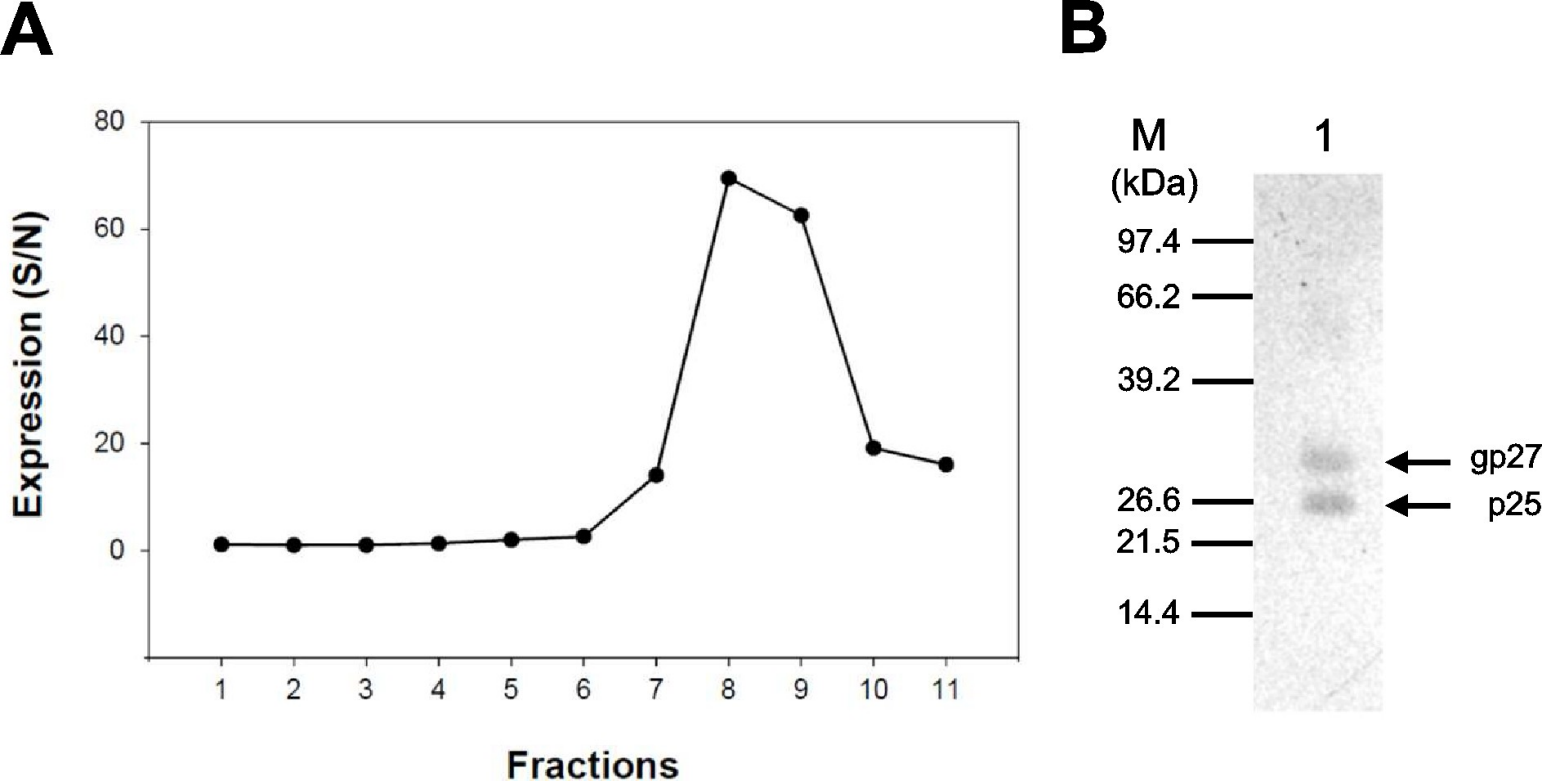
Purification of HBsAg particles by isopycnic kalium bromide (KBr) gradient centrifugation. (A) Cell culture supernatants of primary hepatocytes, which were infected with recombinant vaccinia viruses, were pre-cleared, and proteins were concentrated by trichloroacetic acid (TCA) precipitation prior to separation by isopycnic ultracentrifugation with KBr. Eleven fractions were collected, and the HBsAg in each fraction was detected by an IMx microparticle enzyme immunoassay. (B) Peak fraction was loaded on SDS-PAGE and immunostained with HBsAg-specific goat polyclonal antibodies: M: Premixed protein low range molecular weight standard marker (Boehringer); Lane 1: KBr gradient peak fraction 8. Sizes of reactive proteins are indicated in kilodaltons (KDa).

Primary hepatocytes and the human hepatoma cell line HuH-7, respectively, were seeded in DMEM to express HBsAg. After 2 days of cultivation supernatants were infected with HBsAg recombinant vaccinia viruses. Subsequently, secreted HBsAg was determined by MEIA and subjected to isopycnic KBr ultracentrifugation to purify HBsAg particles. A typical gradient fractionation pattern resulting from a KBr ultracentrifugation of HBsAg particles from human primary liver cells is shown in Fig 3A (Fig 3A). When these fractions were analyzed by silver-stained SDS-PAGE, 2 dominant bands became visible in fraction 8 at a KBr density of 1.2 g/cm^3^ and a molecular weight identical to both the non-glycosylated protein p25 and the glycosylated gp27. The identity of the assumed HBsAg in fraction 8 was confirmed by western blot using HBsAg-specific antibodies (Fig 3B). The purity of this HBs-antigen fraction was >65%, as determined by silver-staining SDS-PAGE.

#### Isocratic column gel filtration

**Fig 4:**
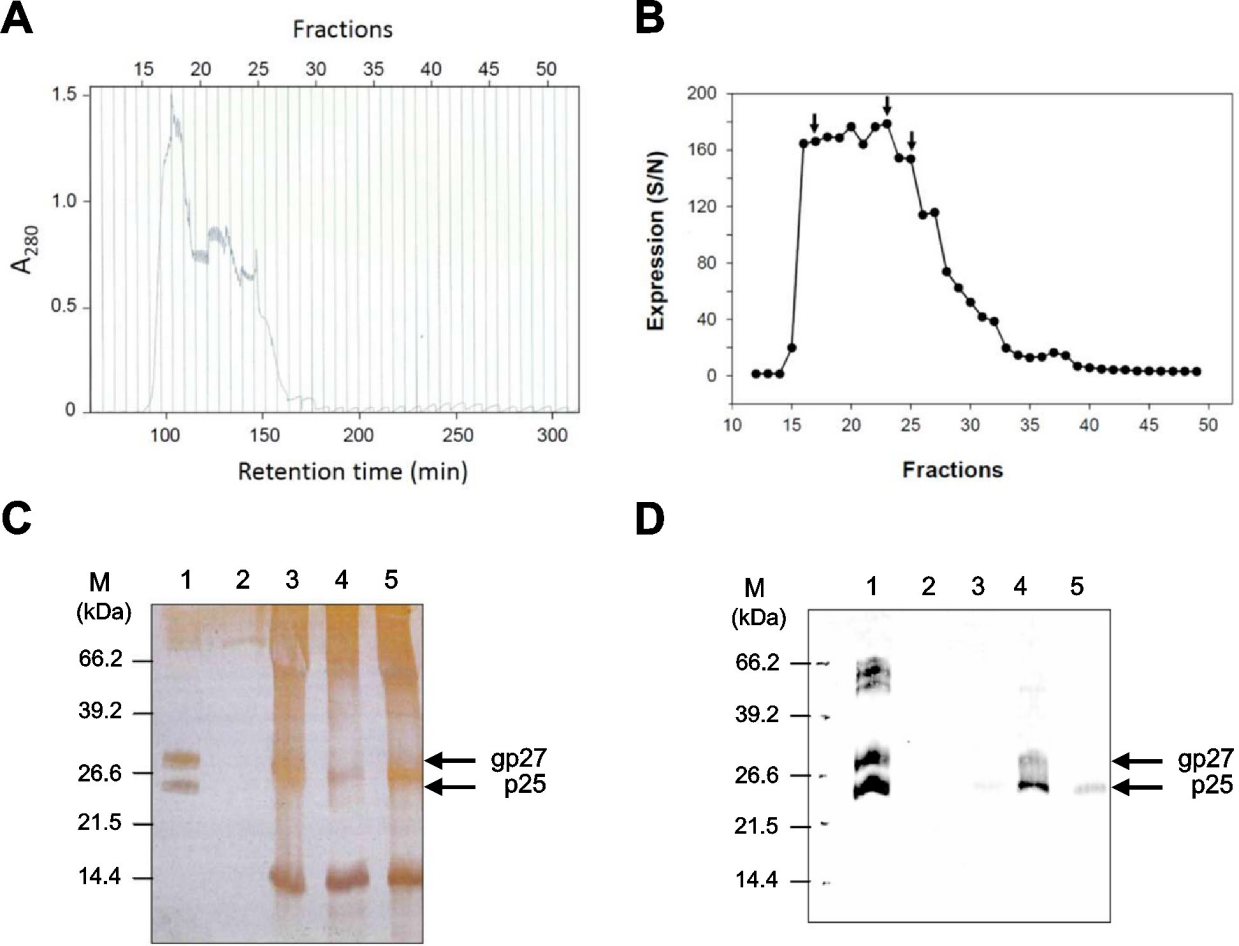
Isocratic column gel filtration. (A) HepG3 protein separation was performed by preparative gel filtration on a Superdex 200 prep grade column. Flow rate was 0.5 ml at a total volume of 120 ml. Flow buffer was PBS (pH 7.5). Sixty fractions of 3 ml each were collected, and the total protein concentration was determined by spectrophotometry at a wavelength of 280 nm. (B) The HBsAg content in collected fractions was determined by a commercial IMx microparticle enzyme immunoassay. (C) Silver-stained (SDS-PAGE) and (D) western blot analysis of selected fractions: Lane 1: Positive control (microparticle purified HBsAg). Lane 2: Fraction 12 as negative control. Lane 3: Fraction 17; Lane 4: Fraction 23; Lane 5: Fraction 25. The highest concentration of HBsAg was found in fraction 23 (Lane 4).

Removal of contaminating proteins and enrichment of HBsAg were the most important purposes of using column chromatography as a purification method. A Superdex 200 prep cell was used as the chromatography medium to separate HepG3-derived HBsAg from contaminating low molecular weight proteins. It was found that under optimal chromatography conditions (flow rate 0.5 ml; total column volume 120ml; PBS flow buffer of pH 7.5), a peak of total protein concentration was determined by photometrical measurement (at 280 nm) between fractions 17 and 18 (Fig 4A). Further, high protein levels appeared as a shoulder from fractions 20 to 25. Analysis of HBsAg in each fraction by MEIA revealed the presence of HBsAg in fractions 16 to 25 with the highest levels at fraction 23 (178.76 S/N) (Fig 4B). Therefore, Superdex 200 prep cell column chromatography was not sufficient to enrich HBsAg fractions from other contaminating proteins. Analysis of the HBsAg-positive column fractions 17, 23, and 25 by SDS-PAGE (Fig 4C) showed highly contaminated HBsAg when compared with other purification methods, such as the anti-HBs-coated microparticles (Lane 1) described later in the manuscript. The abovementioned fractions were tested for HBsAg by western blot analysis (Fig 4D). Fraction 23 showed the highest HBsAg content, coinciding with the results obtained by HBsAg-MEIA determination (Fig 4B).

#### Sucrose cushion

**Fig 5:**
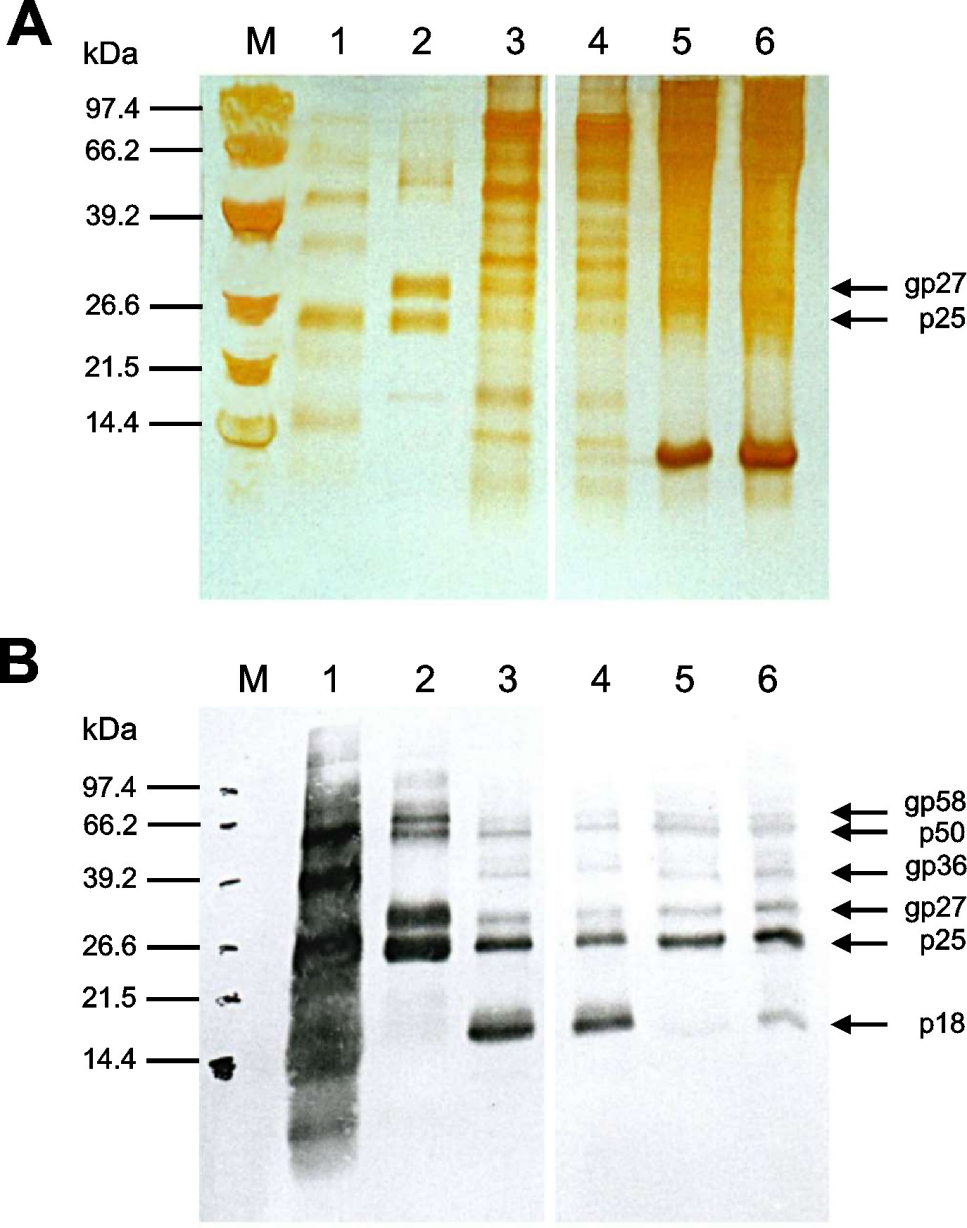
Comparison between HBsAg purified by anti-HBs-coated microparticles and sucrose-cushion purified HBsAg from differently cultured hepatoma-cell-lines. Constitutively HBsAg expressing HepG3-cells were cultured in serum-free and serum-supplemented medium. After 5 days of cultivation HBsAg was concentrated, subjected to 15% SDS-PAGE and analyzed by silver staining (A) or immunoblotting with HBsAg-specific antibodies (B). M: Molecular weight standard; Lane 1: Commercially available human plasma reactive for HBsAg as positive control (Abbott IMx HBsAg Control No. 2228–10); Lane 2: HBsAg purified from recombinant vaccinia virus-infected HuH-7 cells by anti-HBs-coated microparticles (Abbott IMx HBsAg (V2) Reagent Pack No. 2228–21). Lane 3 and 4: Two fractions of HBsAg expressed by HepG3-cells under serum-free conditions. Lane 5 and 6: Two fractions of HBsAg expressed by HepG3-cells in serum-supplemented media. Protein samples were loaded on one gel. Two non-relevant lanes were cut out of the silver-stain (A) and Western-blot (B) images, as indicated. The positions of anti-HBsAg antibody reactive proteins are depicted on the right.

The HBsAg-positive sucrose gradient fractions were combined, dialyzed against PBS, and all insoluble material was removed by low-speed sedimentation. In hepatoma cells, HBsAg-production under serum-free conditions was compared to HBsAg in serum-supplemented media. The cell-line HepG3 was adapted to serum-free cell culture conditions by stepwise reducing the concentrations of fetal calf serum (FCS) up to 0% in order to facilitate and improve the purification of secreted HBsAg particles from cell culture supernatant. Sucrose cushion sedimented HBsAg was suspended in PBS and separated by SDS-PAGE. An examination of this HBsAg probe by silver-stained SDS-PAGE revealed that the final sample still showed high protein contaminations (Fig 5A, Lane 3–6). An explanation for these contaminations might be high amounts of serum albumin, which often interacts with HBsAg. Serum supplemented HepG3-cells (Fig 5A, Lane 5–6) produced approximately 50% higher amounts of HBsAg than HepG3-cells cultivated in serum-free media. In contrast, HBsAg expressed in serum-free media revealed higher purity (Fig 5A, Lanes 3–4). In comparison, the described purification strategy of sucrose cushion was not suitable to obtain sufficient amounts of highly pure HBsAg particles. Specific detection of HBsAg was performed by western blot analysis using goat polyclonal antibodies (Fig 5B). This further revealed that antigens purified from HepG3 cells, especially when adapted to serum-free conditions, included an additional small protein of 18kDa, which was specifically recognized by HBsAg-specific antibodies. This band was only weakly detectable in samples purified from serum-supplemented HBsAg expressing HepG3-cells.

#### Anti-HBs-coated microparticles

The highest amount of glycosylated protein was seen in HBsAg derived from HuH-7 cell expression by the vaccinia system and purified by anti-HBs-coated microparticles. Silver-stained SDS-PAGE analysis of HBsAg expressed in these hepatoma cells revealed purification to near homogeneity (> 98% pure). HBsAg was well separated from contaminating proteins (Fig 5A, Lane 2). HBsAg essentially appeared as both glycosylated (gp27) and non-glycosylated (p25) forms. The identity of the HBsAg was confirmed by western blot analysis using anti-HBsAg antibodies (Fig 5B). In all fractions, dimers of HBs-antigen were found (p50, gp58).

**Fig 6:**
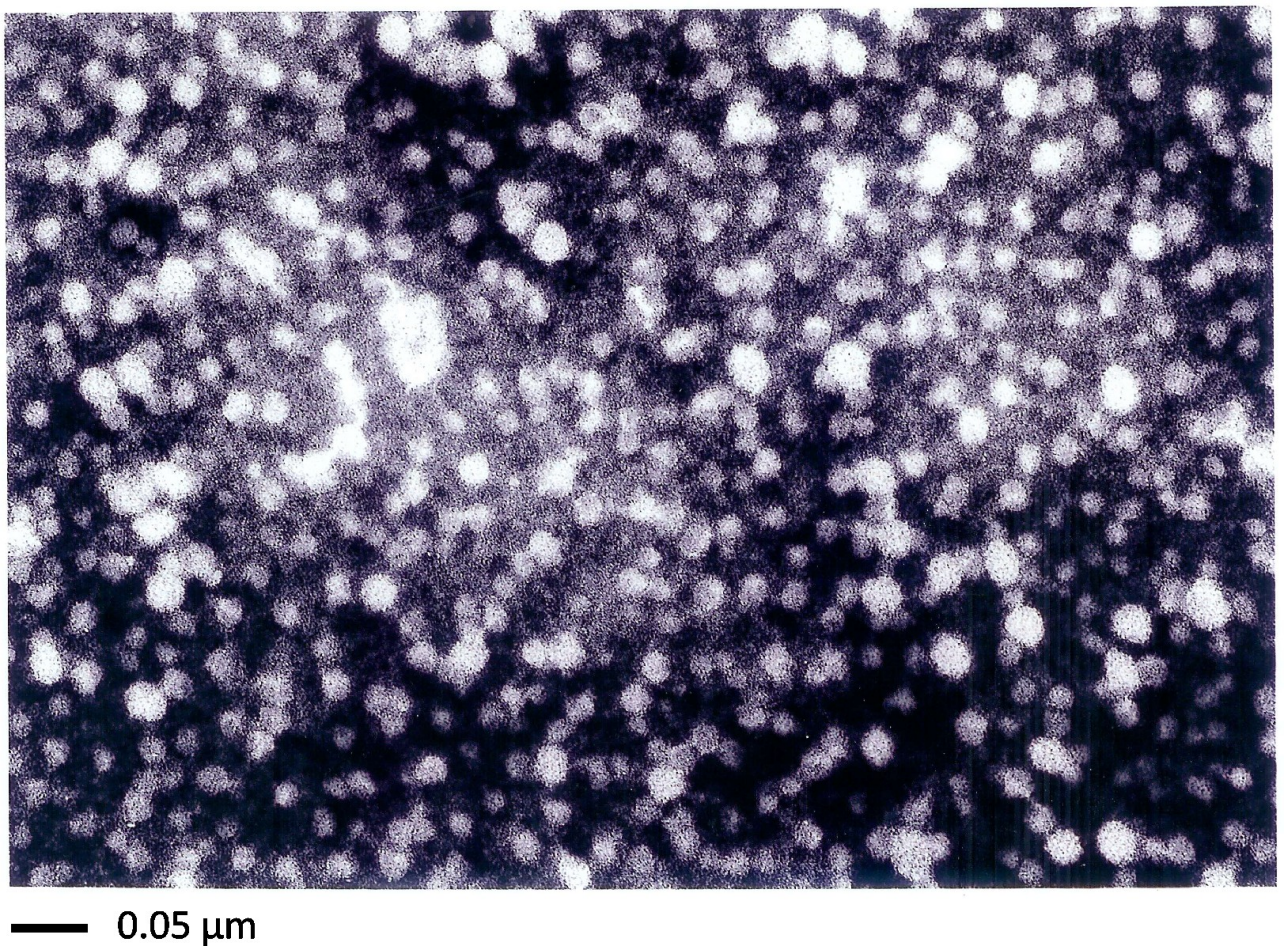
Electron microscopy of microparticle-purified HBsAg particles derived from recombinant vaccinia virus-infected primary hepatocytes. HBsAg particles (22 nm) were negatively stained with 2% aqueous phosphotungstic acid (pH 7.3) and examined by electron microscopy.

A comparison of four different purification methods revealed that the highest degree of HBsAg could be received with anti-HBs-coated-microparticles. Thus, HBsAg purified by this procedure was used as a reference standard for all other analyses in all biochemical assays and immunological tests. Electron microscopy revealed the presence of especially spherical HBsAg particles, heterogeneous in size, ranging from 0.005 to 0.03 μm, with a mean diameter of 0.018 μm (Fig 6). This heterogeneity in size and morphology is in agreement with reports from the literature on the characterization of HBsAg particles either isolated from human plasma or generated *in vitro* [55–58].

## Discussion

In the present research, we analyzed the expression, purification and characterization of HBsAg particles in primary hepatocytes and different human hepatoma cells following infection with recombinant vaccinia viruses. Our results indicated a clear correlation between M.O.I. of the applied vaccinia viruses and the expression efficiency of HuH-7, HepG2, and primary liver cells. To the best of our knowledge, this is the first description of the feasibility of using human primary hepatocyte for the expression of HBsAg using the recombinant vaccinia virus expression system. The highest HBsAg production could be demonstrated in HuH-7-cells infected by recombinant vaccinia viruses. Lower expression rates were found in primary hepatocytes and HepG2-cells. The reasons for the differences in HBsAg expression are not yet well understood. However, the differences in HBsAg-expression could be due to various factors, such as different infectibility of the cells as well as variable cellular factors, which may lead to differences in transcription, translation, and secretion of HBsAg. The human hepatoma cell line HuH-7, which was established from a hepatocellular carcinoma, is known to replicate continuously with good colony growth and doubling time [59]. The human hepatoma cell line HepG2 demonstrated significantly lower expression rates of HBsAg after infection with recombinant vaccinia viruses, which might be due to the fact that these cells possess a membrane-bound receptor that selectively mediates the transport of secretory proteins from the rough endoplasmic reticulum to the Golgi apparatus [60].

The novelty of this paper is the high expression of HBsAg from human primary hepatocytes following infection with HBsAg recombinant vaccinia viruses. Primary hepatocytes infected with high M.O.I. of HBsAg-recombinant vaccinia viruses exhibited high yields of HBsAg production. Primary hepatocytes secreted HBsAg efficiently to the cell culture supernatant with high concentrations of 8 μg/ml (= 8 mg/l), which exceeded, for example, the yield of the total protein extraction of Hepatitis B virus-like M particles from the Saccharomyces cerevisiae strain using alumina powder 8 times [14]. Thus, expression of HBsAg particles by primary hepatocytes following vaccinia viral infection could be an alternative source of S-antigen for further research or diagnostic purposes. Primary liver cells are not genetically modified and possess no oncogenic potential which could be suggested for immortalized liver cell cultures. The results indicate that HBsAg particles were produced in high concentrations and correct post-translational modifications, mainly glycosylation, by HBsAg recombinant vaccinia viruses in both hepatoma cells and primary hepatocytes.

The vaccinia virus system was chosen because it is an excellent expression system. Vaccinia viruses encode their own capping, methylating, and polyadenylating enzymes. The advantage of the system is that the replication of the T7 RNA polymerase is localized in the cytoplasm, and the DNA does not have to enter the nucleus for transcription [61]. HBsAg expression processing and transport to the plasma membrane by vaccinia is therefore possible in all cell lines and in primary human hepatocytes.

Long-term infection of primary hepatocytes by HBsAg-recombinant vaccinia viruses was performed in a double gel configuration. This sandwich configuration resembles more closely the *in vivo* geometry of the cells. In this assay format, the integrity of the cells is maintained, and normal cell-cell communication can be established. Long-term cultivation in human primary hepatocytes was previously mentioned by Ryan et al. [62], who described secretion of albumin by human hepatocytes for more than 2 weeks. This long maintenance of hepatocellular morphology and function in long-term cultures of primary liver cells (> 30 days) was due to the use of a double gel [63]. The data presented here demonstrate that with a culture system that effectively mimics the normal histologic organization of the liver, human hepatocyte morphology and function can be maintained for a prolonged period of time, even following lytic HBsAg-recombinant vaccinia virus infection. The mechanism for this maintenance, however, is unclear. The maximum expression of HBsAg occurred during the first week in culture. Further, in infected lymphocytes, the highest production of HBsAg occurred early after infection using this vaccinia-HBsAg construct [46].

HBsAg used for laboratory research, diagnostics, or vaccination must be free of any contamination by foreign proteins. Therefore, different methods of purification of HBsAg particles were examined and compared, namely isopycnic ultracentrifugation, isocratic column gel filtration, sucrose-cushion sedimentation, and binding by anti-HBs-coated microparticles. The purification by isopycnic ultracentrifugation using KBr allowed removal of cell-contaminating proteins. However, elimination of albumin, which was always found adsorbed to the surface of HBsAg-particles, did not occur during ultracentrifugation. The disadvantage of this method, namely that pre-S components are often destroyed due to high salt effects, was proven in our experiments. By applying the purification method of sucrose cushion sedimentation, which is based on physical characteristics, such as density and particle size of HBsAg, excessive chemical techniques were avoided, but our results proved that the HBsAg sucrose cushion sedimentation step was also not sufficient for the purification of HBsAg. In order to remove serum proteins, such as albumin, from the starting material, the constitutively HBsAg-expressing cell line HepG3 was adapted to serum-free conditions. After purification by sucrose cushion sedimentation, HBsAg produced by cells cultured in serum-free media compared to that in serum-supplemented media showed higher purity, but this increase in purity correlated with the disadvantage of a much lower expression rate of HBsAg under serum-free conditions. Under serum-free conditions, the expression of HBsAg in HepG3-cells led to the appearance of an additional protein of 18 kDa. There is no clear explanation for this observation at present. It may be due to an affected transcription or translation, such as an internal transcriptional stop or the lack of cellular components, such as t-RNAs which lead to a stop in translation.

Our results clearly demonstrate that purification by anti-HBs-coated microparticles revealed the highest degree of purity. For example, albumin, which was regularly fixed closely to the HBsAg-particles and difficult to separate, was easily and totally removed. The pre-S components, which increase the body immune system to protect from HBV infection, are preserved during that purification method. An additional major advantage of that purification method is that it can be carried out in a fast, economical, and simple way. Therefore, this method provides several advantages over other previously described expensive and time-consuming purification strategies, such as precipitation [64,65], affinity chromatography [66,67], and ultracentrifugation [68,69]. Taken together, the combination of different purification methods leads to a maximum in purity, but to a reduced amount of final product. Indeed, the protein yield of HBsAg particles purified by antibody-coated microparticles was about one third of that obtained with the other purification methods. Therefore, an alternative approach would be to combine different column fractionation methods in order to improve protein purity without compromising protein yield.

We found that HBsAg particles made by primary hepatocytes were similar or identical to those obtained by hepatoma cells. Electron microscopy examinations revealed particles with a typical diameter of 22 nm, banding on KBr and sucrose gradients at a density of 1.2 g/cm^3^, which is characteristic of subviral particles. The heterogeneity in size of HBsAg particles isolated from infected primary hepatocytes with anti-HBs-coated microparticles, in particular the smaller size range (<22 nm), might be in part due to the stringent conditions used for particle purification. We cannot exclude that particles have been in part disrupted during purification and reassembled afterward into particles of different sizes, as recently described in *in vitro* disassembly and reassembly assays [57]. It is also possible that, in that process, some particles are incomplete or defective. On the other hand, heterogeneity in size of our HBsAg particles, in particular in the higher size range (>22 nm), is in agreement with various reports from the literature [55–58], and might be in part due to differences in lipid-protein composition. It remains unclear which requirements to the host cell (e.g., the primary hepatocyte and which properties of the small surface antigen) are necessary and responsible for an efficient building and secretion of HBsAg particles. Our purified particles were morphologically, biochemically, and immunologically comparable to HBsAg produced in yeast [70] and other mammalian cells [71] and thus represent attractive alternative S-antigens, which could be used as model systems for laboratory research concerning new diagnostics and HBV vaccines.
